# Quantitative Comparison of Constitutive Promoters in Human ES cells

**DOI:** 10.1371/journal.pone.0012413

**Published:** 2010-08-26

**Authors:** Karin Norrman, Yvonne Fischer, Blandine Bonnamy, Fredrik Wolfhagen Sand, Philippe Ravassard, Henrik Semb

**Affiliations:** 1 Department of Laboratory Medicine, Lund Center for Stem Cell Biology and Cell Therapy, Lund University, Lund, Sweden; 2 Biotechnology and Biotherapy Laboratory, Centre de Recherche de l'Institut du Cerveau et de la Moelle, CNRS UMR 7225, INSERM UMRS 975, University Pierre et Marie Curie, Hôpital Pitié Salpêtrière, Paris, France; Brigham and Women's Hospital, United States of America

## Abstract

**Background:**

Constitutive promoters that ensure sustained and high level gene expression are basic research tools that have a wide range of applications, including studies of human embryology and drug discovery in human embryonic stem cells (hESCs). Numerous cellular/viral promoters that ensure sustained gene expression in various cell types have been identified but systematic comparison of their activities in hESCs is still lacking.

**Methodology/Principal Findings:**

We have quantitatively compared promoter activities of five commonly used constitutive promoters, including the human β-actin promoter (ACTB), cytomegalovirus (CMV), elongation factor-1α, (EF1α), phosphoglycerate kinase (PGK) and ubiquitinC (UbC) in hESCs. Lentiviral gene transfer was used to ensure stable integration of promoter-eGFP constructs into the hESCs genome. Promoter activities were quantitatively compared in long term culture of undifferentiated hESCs and in their differentiated progenies.

**Conclusion/Significance:**

The ACTB, EF1α and PGK promoters showed stable activities during long term culture of undifferentiated hESCs. The ACTB promoter was superior by maintaining expression in 75–80% of the cells after 50 days in culture. During embryoid body (EB) differentiation, promoter activities of all five promoters decreased. Although the EF1α promoter was downregulated in approximately 50% of the cells, it was the most stable promoter during differentiation. Gene expression analysis of differentiated eGFP+ and eGFP- cells indicate that promoter activities might be restricted to specific cell lineages, suggesting the need to carefully select optimal promoters for constitutive gene expression in differentiated hESCs.

## Introduction

Human embryonic stem cells (hESCs) are derived from the inner cell mass (ICM) of the blastocyst and have the unique potential to differentiate to any cell type of fetal and adult tissues [1]. In this sense, hESCs offers an expandable source of in vitro derived human cells that can be used for a wide diversity of applications such as regenerative medicine and cell replacement therapies. However, to fully explore the potential of hESCs, it is important to understand the basic processes that control growth and differentiation of hESCs.

To reveal the molecular pathways behind growth and differentiation of hESCs, efficient genetic engineering techniques are advantageous tools for controlled expression of key regulatory genes or to introduce fluorescent reporter genes such as enhanced green fluorescent protein (eGFP). In these processes, constitutive promoters are useful tools due to their high level of expression in most cell types. The constitutive cytomegalovirus (CMV) enhancer/chicken β-actin promoter (CAG) promoter was recently used for generation of endodermal progenitor cells from hESCs by overexpression of *SOX17* and *SOX7* [2]. To reprogram somatic cells into induced pluripotent cells (iPSCs), the constitutively active elongation factor-1α (EF1α) promoter was used to overexpress the four transcription factors *SOX2, OCT3/4, KLF4* and *c-MYC* [3]–[5]. Moreover, to monitor and track iPSCs generated from mouse embryonic fibroblasts the EF1α promoter was used to constitutively express eGFP [4]. Thereby, continously expressed fluorescent reporter/marker genes holds an emerging promise as tools for live imaging of hESCs in vitro and also for identification of differentiating hESCs in animal grafting experiments without using time consuming species-specific antibody labeling systems or *in situ* hybridization.

Different eukaryotic/mammalian and viral promoters have been reported to efficiently drive expression of transgenes in hESCs. The Envy hESC line expresses eGFP both in undifferentiated cells and in their differentiated progenies as a result of stable integration of a human β-actin promoter(ACTB)-driven eGFP gene [6]. The CMV promoter has been reported to mediate strong expression in various cellular systems but its activity in mouse and human ESCs remains controversial [7]–[9]. The phosphoglycerate kinase (PGK) and the EF1α promoters have also been effectively used for long term constitutive transgene expression in ESCs. Whereas the EF1α- and PGK promoters were shown to mediate stable long term expression of eGFP in hESCs, the CMV promoter only mediated transient expression [10]. Consistently, in mouse ES cells (mESCs), the EF1α and PGK promoters are more stable than the CMV promoter [8]. Additional comparative studies of the CMV and EF1α promoters showed that EF1α is superior to the CMV promoter in undifferentiated mouse, monkey and human ESCs [11]. The EF1α promoter was used to generate stable EF1α-eGFP hESCs that maintained eGFP expression up to four weeks of culture. Furthermore, the mammalian ubiquitinC (UbC) promoter was found to stably drive eGFP expression in hESCs, but at moderate levels compared to the more commonly used CAG promoter [9].

Thus, diverse constitutive promoters have been tested in mouse and human ESCs, but a comprehensive comparison of constitutive promoter activity and stability in undifferentiated and differentiated hESCs is still lacking. For this purpose, we performed a comparative study of the activities of the ACTB, CMV, EF1α, PGK and UbC promoters in hESCs. Lentiviral mediated gene transfer was chosen as gene delivery system since it is known to efficiently introduce genetic material into the hESC genome [12], [13]. In addition, compared to traditional retroviral vectors, lentiviral gene expression is maintained during propagation and differentiation of embryonic stem cells [14]. Other viral systems, such as adenovirus have been used for gene delivery into hESCs but since they usually do not integrate their genome into the host chromosomes, transgenes can only be transiently expressed [15], [16]. The constitutive promoters were cloned into lentiviral self-inactivating vectors that lack endogenous promoter activity from the long terminal repeats. Transcription of an eGFP gene present in the lentiviral vectors was therefore solely driven by the introduced constitutive promoters. Promoter activity was monitored by the expression of eGFP in long term culture of undifferentiated hESCs and in cells differentiated into all three embryonic germ layers. Our data demonstrate that ACTB and PGK promoters mediated stable transcriptional activity resulting in high levels of transgene expression in long term culture of undifferentiated hESCs. Transcriptional activities of all five promoters were downregulated during differentiation of hESCs. Notably, despite this downregulation, some promoters sustained reporter gene expression in a germ layer-specific manner.

## Results

### Lentiviral transduction and gene copy number determination

**Figure 1:**
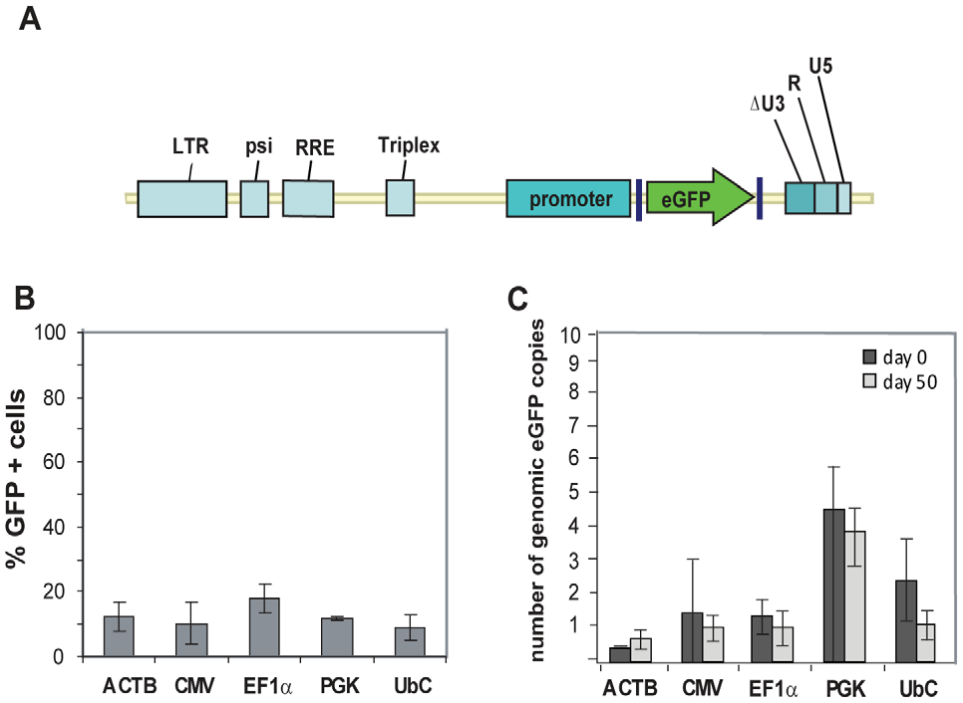
Transduction efficiency in hESCs SA121. **A**. Schematic representation of pTRIP lentiviral vectors, in which eGFP are under the control of ACTB-, CMV-, EF1α-, PGK- or UbC promoters. The hESC line SA121 was transduced with lentiviral vectors; pTRIP-ACTB-, CMV-, EF1α-, PGK-or UbC-eGFP. Ten days after transduction, cell populations were analyzed by FACS to determine the percentage of cells that expressed GFP. **B**. Percentage of transduced hESCs expressing eGFP. **C**. Determination of number of transgenic inserts in the GFP positive cells by qPCR at time of FACS isolation (day 0) and after 50 days of culture. Data in A and B are shown as mean of three independent experiments. Error bars represent standard deviation of the mean (±s.d.).

The hESC line SA121 was transduced with ACTB-, CMV-, EF1α-, PGK- and UbC-eGFP self-inactivating lentiviral vectors (Fig. 1A). Efficiency of transductions was measured by flow cytometry (FACS) as percentage eGFP+ cells. To be able to quantitatively compare eGFP expression between the different promoters we aimed for similar copy numbers of integrated viral vectors. In addition, to avoid insertional mutagenesis, we transduced hESCs with low vector to target cell ratios. Initial test transduction experiments revealed that multiplicity of infection (MOI) 1 would generate transduction efficiencies up to 35% eGFP+cells for hESC line SA121 for the CMV-, EF1α and PGK-eGFP lentiviral vectors (Fig. S1A). Based on these experiments, hESC line SA121 was transduced at MOI 1 with ACTB-, EF1α-, CMV-, PGK and UbC-eGFP lentiviral vectors, which generated a maximum of 20% eGFP+ cells (Fig. 1B). Previous reports have showed that nonintegrated lentiviral vectors transiently expressed the transgene up to 10 days after transduction and thereafter gradually decreased as a result of dilution of the vector genome through cell divisions. [17], [18]. Therefore, transduction efficiencies were analyzed 10 days after transduction in order to avoid detection of transgene expression from nonintegrated vectors. qPCR on genomic DNA from FACS-isolated eGFP+ cells demonstrated that on average 1–2 viral vector copies per eGFP+ cell were integrated in ACTB-, CMV-, EF1α- and UbC-eGFP cells (Fig. 1C). PGK-eGFP transduced cells contained approximately 5 vector copies per eGFP+ cell. After 50 days of culture of eGFP+ isolated cells, copy numbers were detected at similar levels as immediately after FACS isolation with the exception of UbC promoters that decreased from average 2 vector copies to 1 copy per eGFP+ cell (Fig. 1C). Average gene copy numbers were measured by comparing the amount of *eGFP* and *CDX2* amplified PCR products. Generation of a standard curve verified linear amplification of *eGFP* and *CDX2* genomic DNA amplicons at similar efficiency (Fig. S1F).

### Promoter activity in undifferentiated hESCs

**Figure 2:**
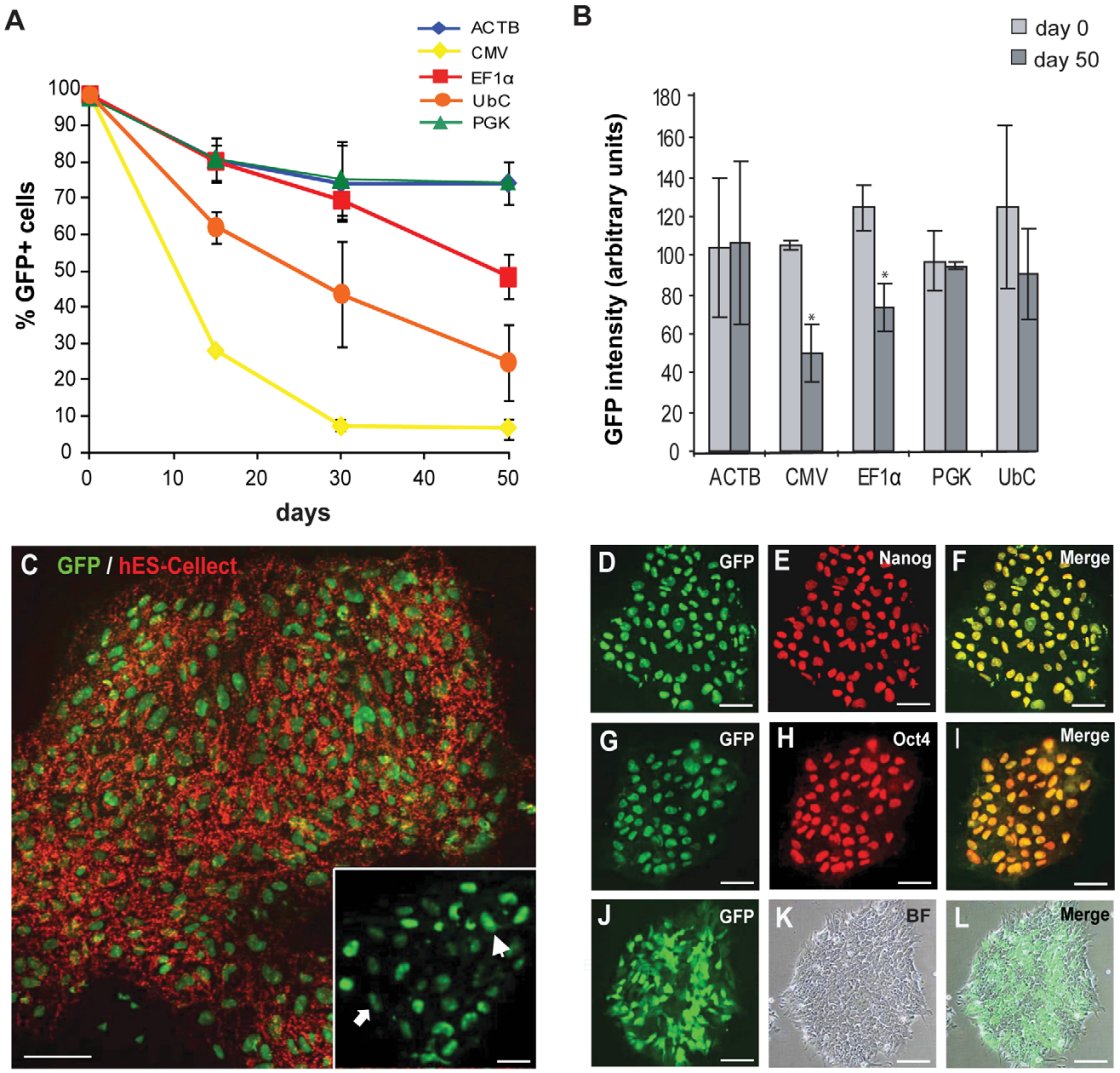
Constitutive promoter activity in long term culture of undifferentiated hESCs. The hESC line SA121 was transduced with lentiviral vectors containg the pTRIP-ACTB-, CMV-, EF1α-, PGK-or UbC-eGFP lentiviral vectors. 10 days after transduction, eGFP+ and eGFP− cells were separated by FACS sorting, referred to as day 0. Isolated eGFP+ cells were thereafter cultured for 50 days under self-renewing conditions and promoter activities were measured by FACS analysis at day 0, 15, 30, and 50. **A**. Promoter activities as percentage of eGFP+ cells at day 0, 15, 30 and 50. **B**. Intensity of fluorescent signal of eGFP expression from the same eGFP positive cells that were FACS analysed day 0 and 50. Intensity was measured by FACS analysis and EF1α promoter showed a significant decrease in intensity of eGFP expression from day 0 to day 50 (*p<0,014 students t'test). Decrease of ACTB-, CMV-, PGK- and UbC-eGFP intensity from day 0 to day 50 is not significant. **A**–**C**. Data are shown as mean of three independent experiments. Error bars represent standard deviation of the mean (± s.d.). **C**–**L**. Immunofluorescence stainings of ACTB-eGFP+ cells 30 days after FACS sorting. eGFP expressing cells show uniform expression of pluripotency markers; hES-Cellect (**C**), inset in C shows low and high intensity eGFP expressing cells, Nanog (**D**–**F**) and Oct3/4 **(G**–**I**). **L**. Merged image of colony morphology of human ES cells cultured on Matrigel (**K**) and eGFP expression within the colony (**J**). Scale bar in **C** represent 100 µM, inset and **D**–**L** 200 µM. Cells representative of high eGFP expressing is indicated by arrowhead and low eGFP expression by arrow.

To be able to quantitatively compare the activity of the different promoters, we isolated eGFP+ cells from the promoter-eGFP transduced populations by FACS sorting. Results for promoter-eGFP transduced populations are representative of three independent transductions that were FACS sorted separately and eGFP+ isolated cells were maintained as separate cell cultures in order to exclude possible variation in transductions or maintenance of cells. eGFP+ sorted cells exhibited characteristic hESC morphology and uniform expression of pluripotency markers OCT3/4, NANOG, and hES-Cellect (Cellartis AB) when cultured on Matrigel in mTESR cell culture medium, confirming that the transduction and FACS-sorting procedure did not affect hESC pluripotency (Fig. 2C–L). Promoter activity was measured 15, 30 and 50 days after FACS sorting and the sorting day was referred to as day 0. Percentage eGFP+ at day0 was approximately 98% for all promoters since reanalysis of sorted cells showed a purity of 97–100% eGFP+ cells (Fig. 2A and Table 1). The ACTB-, EF1α- and PGK promoters were found to be more efficient than CMV in driving long term expression of eGFP. In particular, the ACTB and PGK promoters mediated sustained eGFP expression (74,0±5,8% and 74,4±10,6%) after 50 days in culture (Table 1). Equal promoter activities were detected for ACTB, EF1α and PGK up to day 30 but thereafter EF1α activity decreased. The percentage of CMV-eGFP+ cells decreased to 6,7±2,9% at day 50. Already at day 7, the CMV promoter was rapidly downregulated and was expressed in approximately 30% of the cells (data not shown). Activity of the UbC promoter was observed in 62±4,5% of the total cell populations at day 15 and decreased to 24,8±10,4% after 50 days of culture (Fig. 2A and Table 1). At day 50, promoter activities for ACTB, EF1α and PGK were significantly higher than for the CMV promoter (p≤0.0001 students t'test).

**Table 1:** % eGFP+ cells of hESC line SA121 transduced with pTRIP-ACTB-, CMV-, EF1α-, PGK or UbC-eGFP lentiviral vectors.

Days	ACTB	CMV	EF1α	PGK	UbC
**0**	98±1	98,3±0,6	98±1,5	97,7±0,6	98,3±0,6
**15**	80,5±6,1	27,9±7,1	79,9±4,8	80,6±14,4	62,0±4,5
**30**	74,0±10,5	7,1±0,7	69,3±4,2	75,0±3,8	43,6±14,3
**50**	74,0±5,8	6,7±2,9	48,3±6,1	74,4±10,6	24,9±10,4

Data are shown as mean of three independent experiments ± s.d.

To evaluate the strength of each promoter during maintenance of hESCs, the intensity of the eGFP fluorescent signal was compared at day 0 and day 50 (Fig. 2B). All promoters mediated eGFP expression with similar intensity on day0. After 50 days, the ACTB and PGK promoters maintained high eGFP expression levels, whereas expression levels dropped with CMV, EF1α and UbC and promoters.

To verify that the measured promoter activities were not specific to hESC line SA121, we repeated transduction experiments in hESC line Hues-4 [19], [20]. Transduction at MOI 1 resulted in low copy number integration and transduction efficiencies below 40% (Fig. S1A–C). The stability of the five constitutive promoters in Hues-4 was similar to that measured in SA121 (Fig. S1D). Thus, the ACTB-, EF1α- and PGK promoters maintained sustained activity up to day 30 (86,5±0,1, 80,0±10,1and 76,0±1,0% eGFP+ cells) whereas CMV promoter activity deceased strongly within 15 days (Table S1 and Fig. S1D). However, between day 30 and 50, the PGK promoter activity decreased more in Hues-4 than in SA121 (compare Fig. S1D with 2A). Like in SA121, activities at day 50 were significantly higher for ACTB-, EF1α- and PGK promoters than for CMV (p≤0.001 students t'test). In addition, intensity of the eGFP expression was comparable at day 0 for all promoters and showed similar pattern of stability as measured in SA121 (Fig. S1E). These observations reveal that the relative promoter stability and activity data are comparable between the two tested cell lines.

In summary, of all the tested constitutive promoters the EF1α, PGK and ACTB promoters were the most stable. The ACTB promoter was the most superior promoter in undifferentiated hESCs by maintaining transgene expression in 75–85% of the cells after 50 days in culture in both cell lines tested. In addition, the PGK promoter was found to express eGFP at high intensity up to 50 days of culture, whereas the intensities from the other promoters decreased to various extents.

### Promoter activity in differentiated hESCs

**Figure 3:**
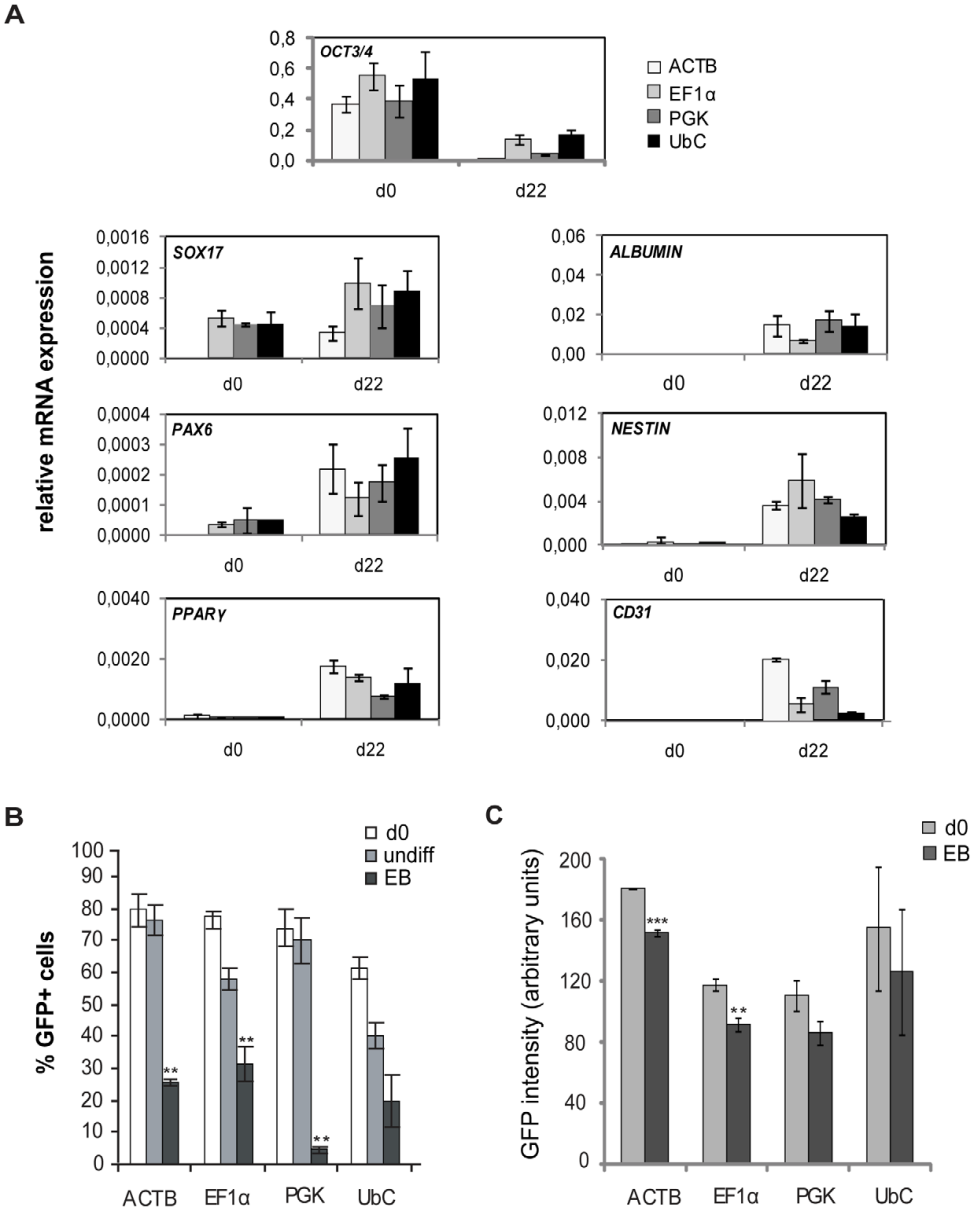
Promoter activity during differentiation of hESCs. **A.** Gene expression analysis of undifferentiated ACTB-, CMV-, EF1α-, PGK- or UbC-eGFP transduced hESCs and after EB differentiation, plotted as relative to reference gene *GAPDH. SOX17, ALBUMIN, PAX6, NESTIN, PPARγ* and, *CD31* were used as marker genes for endodermal, ectodermal and mesodermal cell lineages and OCT3/4 as pluripotency marker. Results in A–C are plotted as mean of three independent experiments and error bars indicate ± s.d, **B**. hESC line SA121 was differentiated as embryonic bodies for 22 days and promoter activities in ACTB-, CMV-, EF1α-, PGK- or UbC-eGFP transduced cells were measured by FACS analysis as % eGFP+ cells. In parallel, % eGFP+ cells were measured on ACTB-, CMV-, EF1α-, PGK- or UbC-eGFP transduced cells that were maintained in their undifferentiated state for 22 days. Statistical analysis of EB day 22 as compared to undifferentiated day 22 (**p≤0.0039 students t'test). **C.** Average level of intensity of eGFP fluorescent signal of the eGFP+ population, detected by FACS analysis at start of differentiation, day 0 and after 22 days, measured as average mean fluorescence intensity. Statistical analysis of EB d22 as compared to undifferentiated cells day 0 (**p<0.001,***p<0.0001 students t'test).

To test the effectiveness of the promoters in differentiated hESCs, eGFP+ sorted cells were differentiated as embryoid bodies for 22 days. Differentiation into cell lineages of all three embryonic germ layers was verified by quantitative real time PCR (qPCR) showing an increase of gene expression levels of endodermal, mesodermal and ectodermal markers genes (Fig. 3A). In addition, the expression level of the pluripotency marker *OCT3/4* decreased. FACS analysis performed at the end of differentiation demonstrated that promoter activities were less stable than in undifferentiated hESCs (Fig. 3B). EF1α was the most stable promoter during differentiation. Nevertheless, it was significantly downregulated during differentiation and was inactive in approximately 50% of the differentiated cells. In contrast to the stable PGK activity detected in undifferentiated cells, its activity was significantly downregulated during differentiation. The CMV promoter was active in only a small portion of the cells (approximately 15%) at start of the differentiation and was therefore not included in differentiation studies.

To evaluate the strength of each promoter during EB differentiation, intensity of eGFP fluorescent signal was measured by FACS analysis of eGFP+ cells. During EB differentiation, intensity of eGFP expression of ACTB- and EF1α was significantly reduced, whereas eGFP fluorescent signal within the PGK- and UbC-eGFP+ populations did not decrease during differentiation (Fig. 3C).

**Figure 4:**
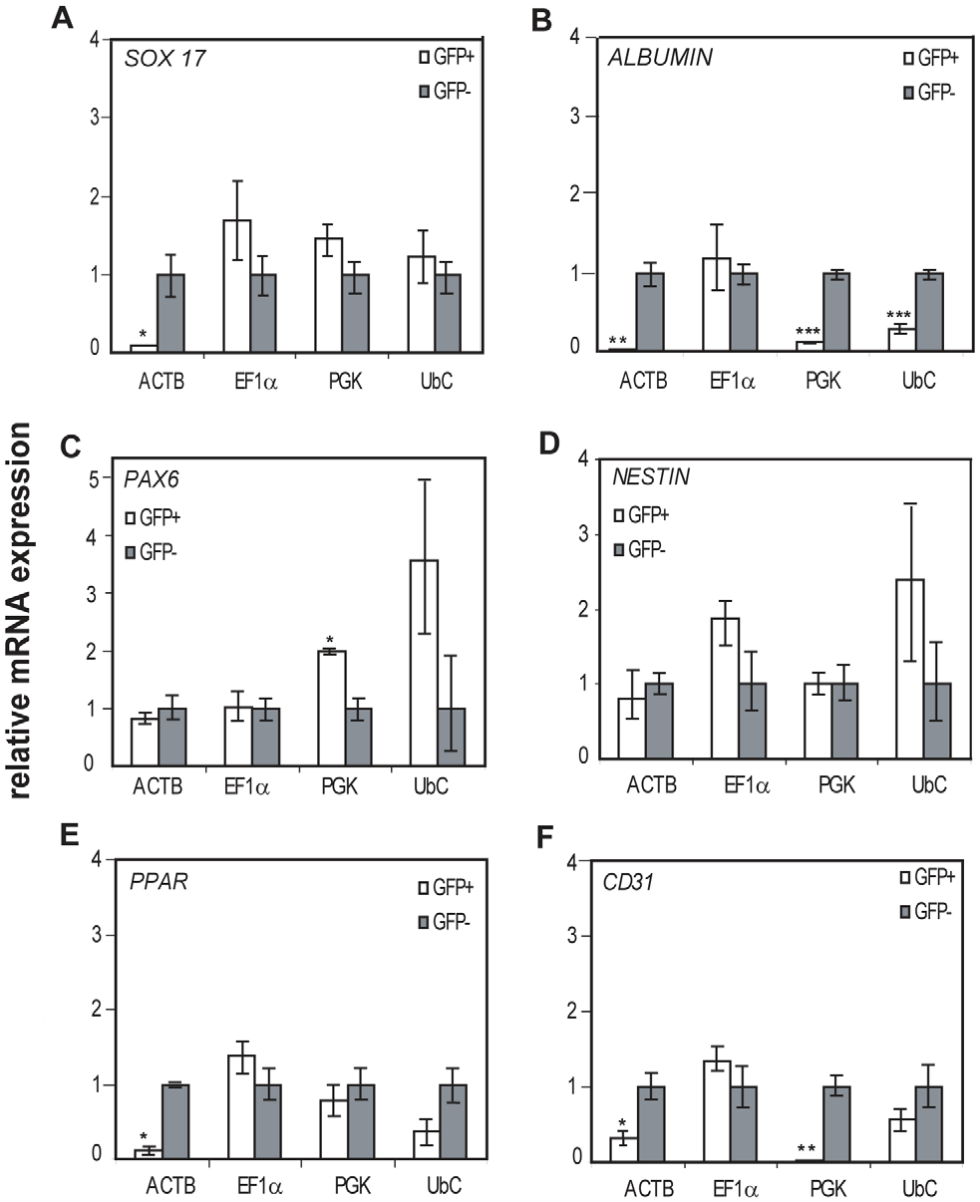
Activity of promoters in cell types representing all three embryonic germ layers. hESCs were spontaneously differentiated for 22 days and thereafter separated by FACS sorting into the eGFP+ and eGFP− cell populations. Relative gene expression was performed by qPCR on the eGFP+ and eGFP− populations. **A**–**F.** Expression analysis of genes representative for differentiation to the three embryonic germ layers; endoderm, mesoderm and ectoderm. *SOX17*(**A**) and *ALBUMIN*(**B**) originates from endoderm. Neural progenitors *PAX6* (**C)** and *NESTIN* (**D**) originates from ectoderm. E–F. Mesodermal cells; PPAR*γ* (**E**) and *CD31* (**F**). Expression levels for each gene in eGFP+ and eGFP− populations are plotted as relative to expression levels in undifferentiated hESCs. Results are plotted as mean of three independent experiments and error bars indicate ± s.d. Statistical analysis of GFP+cells compared to GFP− cells determined by students t'test (p*≤0,0392).

The observation that promoter-mediated transgene expression was downregulated during differentiation raised the question if promoter activities could be restricted to specific cell lineages. To address this, differentiated cells were separated into eGFP+ and eGFP-populations by FACS sorting. Gene expression analysis of eGFP+ and eGFP− populations were carried out for quantification of mRNA levels of marker genes representing the three embryonic germ layers. Notably, in cells where the EF1α promoter is active, mRNA expression levels of genes representing all three embryonic germ layers were similar to or higher than the levels in eGFP− cells (Fig. 4). In addition to these data, FACS analysis showed that the EF1α promoter was less prone to downregulation compared to other promoters during in vitro differentiation (Fig. 3B).

*SOX17*, which is expressed in the primitive streak and endoderm, is expressed at similar levels in EF1α -, PGK- and UbC-eGFP+ cells compared to eGFP− cells implying that these promoters are active during mesendoderm differentiation (Fig. 4A). In contrast, *SOX17* expression was significantly reduced in ACTB-eGFP+ cells compared to ACTB-eGFP− cells. The hepatoblast marker *ALBUMIN* was expressed at significantly lower levels in the ACTB-, PGK- and UbC-eGFP+ populations compared to GFP−cells, suggesting that these promoters are inactive in hepatoblast cells (Fig. 4B).

In cells where the ACTB, EF1α, PGK or UbC promoters were active, higher or equal mRNA levels for neural marker genes *PAX6* and *NESTIN* were detected compared to GFP− cells, indicating that all four promoters are active in neural progenitor cells (Fig. 4C–D). *PPARγ* is expressed in adipose tissues of mesodermal origin. Both the EF1α- and the PGK promoter exhibited similar mRNA expression levels of *PPARγ* in eGFP+ vs eGFP− cells, suggesting that these promoters are active in the *PPARγ+* cells (Fig. 4E). However, ACTB-eGFP+ cell populations showed significantly lower levels of *PPARγ* expression than GFP− cells, indicating lower activity of ACTB promoter in *PPARγ*+ cells. Gene expression analysis of *CD31*, a marker for endothelial cells was significantly lower in ACTB and PGK-eGFP+ populations, suggesting that these promoters are inactive in endothelial cell types (Fig. 4F). In contrast, EF1α-eGFP+ cells showed similar levels of *CD31* expression as EF1α -eGFP− cells, suggesting that the EF1α promoter efficiently drives eGFP expression in endothelial cells (Fig. 4F).

## Discussion

### Promoter activity in undifferentiated hESCs

A number of different constitutive promoters have successfully been reported to maintain stable transgene expression in hESCs and are therefore good candidates in applications like cell lineage tracing, generation of fluorescent reporter cell lines and overexpression of transcription factors. Much effort has focused on techniques for stable integration of transgenes, but attempts to quantitatively compare the effectiveness of constitutive promoters to monitor and track cell fate determination in differentiating hESCs are limited. Here, we quantitatively compare the efficiency of ACTB, CMV, EF1α, PGK and UbC promoters to constitutively drive eGFP expression both in undifferentiated cells and in their differentiated progenies. To achieve this, we applied lentiviral gene delivery to ensure high transduction efficiency and stable transgene integration into the hESC genome. We reasoned that the lentiviral system was more favorable for stable integration of the transgene, than other approved transgene delivery methods in hESCs such as transfection or adenoviral transduction, which are more appropriate for transient expression [15], [16], [21], [22]. Moreover, HIV-1-derived lentiviral vectors are efficient tools for stable genetic modification of mammalian ES cells, since they are less prone to silencing than traditional retroviral vectors [12], [23]–[26]. Here, we provide relative data on promoter characteristics in hESCs using eGFP as reporter gene and thus results presented here are representative for constitutive promoter activities detected as eGFP expression. Nevertheless, it cannot be excluded that possible interference between the promoter and reporter gene might influence transgene expression and therefore future studies will have to resolve if different promoter/reporter combinations will result in other expression and stability profiles than those reported here.

The ACTB promoter was found to be the most stable promoter mediating stable transgene (eGFP) expression during long term culture (50 days) of undifferentiated hESCs. These observations were observed in two independent hESC lines. This promoter has previously been reported to stably express eGFP in both undifferentiated cells and derivatives of all three embryonic germ layers when inserted into to the Envy locus by transfection of bacterial plasmids[6]. Here, we used lentivirus as gene delivery tools for random insertion of the transgene and our data confirm that the ACTB promoter has the potential to generate sustained high level transgene expression in long term culture of undifferentiated hESCs.

The percentage of eGFP+ cells in the EF1α transduced populations decreased after 30 days in both hESC lines. Previous publications suggest that the EF1α promoter acts as a strong and stable promoter for transgene (eGFP) expression in hESCs [11]–[13]. Thus, the EF1α promoter has been used to generate stable eGFP expressing hESC lines with 95% of the cells maintaining eGFP expression up to four weeks [11]. This is in line with our data demonstrating stable eGFP expression up to 30 days in culture. In another study, hESCs transduced with low viral vectors copy numbers showed sustained high EF1α promoter activity for up to 60 days in culture [13]. After 30 days the EF1α promoter activity declined, albeit to a lower degree compared to in our study. The observed difference in EF1α promoter activity during long term culture of undifferentiated hESCs may be explained by lentiviral vector design or differences related to hESC culture techniques.

The percentage of eGFP+ cells in the PGK transduced populations decreased after 30 days in one of the two hESC lines studied. The PGK promoter has not been extensively studied in hESCs and quantitative information about its activity in relation to other constitutive promoters is lacking. Therefore, further studies are needed to elucidate long term activity of the PGK promoter in undifferentitated hESCs and possible variation of PGK promoter activity between hESC lines.

In both cell lines, the UbC and CMV promoters experienced a pronounced downregulation after 50 days in culture. This is consistent with other reports demonstrating substantial loss of UbC driven transgene expression in hESCs [9]. The rapid downregulation of CMV promoter activity during long term culture of undifferentiated hESCs is consistent with recent findings demonstrating that the CMV promoter is not stably expressed in undifferentiated mammalian ES cells [11], [27]. Furthermore, difficulties in obtaining stable CMV-eGFP expressing hESC lines support the inability of the CMV promoter to sustain stable and efficient transcriptional activity in undifferentiated hESCs [9], [11]. Thus, of the analyzed promoters the UbC and CMV are the least stable promoters during long term culture of undifferentiated hESCs (Fig. 2A and S1).

In the present study, analysis of transgene expression is performed on pools of transfected cells rather than isolated subclones. Therefore, it could be hypothesized that loss of transgene expression is caused by selective growth or survival of subclones with low expression levels of the transgene. However, we suggest that any negative effect of high transgene expression on hESC growth and survival would manifest itself equally in all promoter-eGFP transduced hESC cultures, rather than acting only on certain promoter-eGFP transduced cultures but not on others. Moreover, vector copy numbers remained constant throughout the 50 days culture period for all promoters except for the UbC promoter (Fig. 1C). Therefore, we propose that the variations in promoter stability reported here are mainly caused by promoter-dependent variations in transgene expression rather than variations in copy number and integration site in individual cells.

To successfully use reporter cell lines to follow and track cells it is important not only to ensure stable activity of the promoter but also to rely on strong promoter activity. The latter is necessary to ensure detectable levels of reporter gene expression. Thus, the fluorescent signal detected from ACTB-, CMV-, EF1α-, PGK- and UbC-eGFP expressing cells were used to assess strength of the different promoters. At day 0, eGFP levels were expressed at similar intensity for all five promoters. Notably, the ACTB and PGK promoters expressed eGFP at stable intensity levels during the 50-day observation period in both analyzed hESC lines (Fig. 2B and S1E), suggesting that these promoters ensures stable levels of transgene expression. In addition, the ACTB promoter stayed active in the majority of transduced cells over time, indicating that this is a strong and stable promoter in undifferentiated hESCs.

### Promoter activity in differentiated hESCs

Ectopic expression of key regulatory genes is an important tool to study mechanisms of differentiation and to induce cell fate specification in hESCs. This emphasizes the need to identify constitutive promoters that remains active at high and stable levels not only in undifferentiated hESCs but also in their differentiated progenies.

Here, we provide quantitative data demonstrating that the activities of commonly used constitutive promoters decrease during hESC differentiation to various extend, whereby the EF1α promoter showed highest stability. Few attempts have been made to quantify constitutive promoter activity during EB differentiation of hESCs but it has been reported that in EF1α-eGFP transduced hESCs, the number of eGFP+ cells decreased from 84% to 78% eGFP+ cells during 4 weeks of EB differentiation [12]. During EB differentiation of mESCs, EF1α was shown as a superior promoter compared to the PGK promoter [8]. Moreover, during EB differentiation of EF1α-eGFP transduced mESCs, eGFP expression remained stable as observed by fluorescence microscopy, although quantitative qPCR analysis showed that eGFP mRNA levels decreased by approximately 40% [27].

HIV-1 based lentiviral vectors are known to be more efficiently expressed than their MLV gammaretrovirus counterparts that are often transcriptionally silent in both ES cells and in transgenic animals [26], [28]. Silencing have mainly been studied for retroviral vectors and encompasses several related phenomena including complete transcriptional silencing, which is observed shortly after infection, and variegation. The latter refers to the situation when genetically identical sister cells that inherit the same provirus either express or silence the provirus [29]–[31]. Finally, extinction refers to the progressive silencing of an initially expressed provirus during long-term culture or EB differentiation of mESCs [23], [30]. Little is known about silencing of SIN lentiviral vectors in ES cells but it has been shown that transgenes are efficiently expressed at multiple copy integrations but single copy integrations results in inconsistent expression [12], [13], [23], [28]. Detailed examination of mESC clones with single copy SIN lentivirus integrations suggests that lentiviral vectors are silenced by similar epigenetic modifications as their retroviral counterparts [32]. However, since we observed promoter-specific differences in the degree of eGFP inactivation, we conclude that the stability of lentiviral-mediated transgene expression in differentiating hESCs is at least partly dependent on the applied promoter.

Gene expression analysis of eGFP+ and eGFP− separated cells revealed that marker genes characteristic for the three germ layers were expressed in equal levels in EF1α-eGFP+ and eGFP−cells (Fig. 4). Thus, we conclude that activity of the EF1α promoter does not show any preference to endodermal, mesodermal or ectodermal hESC derivatives. The EF1α, PGK and UbC promoters were active in cells differentiating towards ectodermal lineages and in *SOX17*+ early endoderm. Notably, the EF1α promoter remained active during later stages of differentiation, here marked as *ALBUMIN+* late endoderm/hepatoblast cells and in *PPARγ+* and *CD31+* late mesoderm while the ACTB promoter was not active in these populations.

In summary, the ACTB, EF1α and PGK promoters were the most stable promoters in terms of maintaining transgene (eGFP) expression during long term culture of undifferentiated hESCs. Furthermore, the intensity of eGFP expression from the ACTB and PGK promoter were expressed at stable levels during long term culture, whereas the intensities of eGFP expression from the other promoters decreased to various extents. In addition, our data demonstrate that during hESC differentiation, expression of constitutive promoters may be restricted to specific cell lineages and careful selection of promoters is thus important to ensure high transgene expression in differentiated hESC progenies. Our data provides a guideline to choose a suitable promoter to obtain stable gene expression in undifferentiated hESCs and when in vitro differentiation to certain germ layers is desired.

## Materials and Methods

### Culture of human embryonic stem cells

The hESC lines SA121 (Cellartis AB), previously adapted to enzymatic dissociation, and Hues-4 (D.A Melton, Howard Hughes Medical Institute, Harvard Institute, Cambridge, MA) were cultured according to protocols at http://www.mcb.harvard.edu/melton/HUES/ as previously described on mitotically inactivated mouse embryonic feeder cells (Lund Transgenic Core Facility, Lund University, Sweden) [19], [20].

eGFP+ cells were transferred to feeder free culture conditions using Matrigel Matrix (BD Biosciences) in mTESR.1 cell culture medium (Stemcell Technologies) according to manufacturer's instructions, and passaged every fifth to sixth day at 1∶3 split ratio.

For spontaneous differentiation of embryoid bodies, cells were dissociated with 0,05% tryspin-EDTA (Gibco) and cultured as suspension cultures in Knockout-DMEM (Gibco) supplemented with 20% Knockout-serum replacement (Gibco), 1% Non-essential amino acids (Gibco), 1% Glutamax (Gibco), 0.1% beta-mercaptoethanol (Gibco), 1% penicillin-streptomycin (Invitrogen) for 22 days with medium change every third day.

Cells were karyotyped by standard G-banding at Divison of Clinical Genetics, Linkoping University and Lund University, Sweden. SA121 were found to be karyotypically normal and Hues-4 was normal in 60% of the analyzed cells.

### DNA constructs and recombinant lentiviral production

The backbone of the lentiviral construct, pTRIP, has been previously described [33]. The vector, pTRIP ΔU3.CMV-eGFP and pTRIP ΔU3.PGK-eGFP expresses the *eGFP* gene under the control of an internal cytomegalovirus (CMV) promoter and mouse phophoglycerate kinase promoter respectively kindly provided Alexis Pierre Bemelmans [34]. New lentiviral vectors pTRIP ΔU3.ACTB-eGFP, pTRIPΔU3.EF1α-eGFP and pTRIPΔU3.UbC-eGFP were constructed using the Gateway in vitro recombination system (Invitrogen). Briefly the RIP405 promoter was removed by MluI and BamHI restriction from pTRIP ΔU3.RIP405-eGFP [35]. Both extremities were filled by klenow polymerase and the RFA Gateway cassette was cloned to generate the pTRIP ΔU3.RFA (Gateway)-eGFP destination vector. All promoters were cloned by PCR into Gateway compatible Entry clones and finally inserted into the destination lentiviral vector by LR Clonase II recombination according to manufacturer's recommendations (Invitrogen). The elongation factor-1α (EF1α) promoter was amplified from vector pLOX/EWgfp (kindly provided by Dr. S. Karlsson Dept of Molecular Medicine and Gene Therapy, Lund University, Sweden) with primers forward 5′ GGGGACAAGTTTGTACAAAAAAGCAGGCT′3 and reverse 5′GGGGACCACTTTGTACAAGAAAGCTGGGTACTTTGAACCACTGTCTGAGGCTT ′3. The resulting PCR product was recombined into pDONR201 (Invitrogen) to generate EF1α entry clone. The human beta actin promoter (ACTB*)* was amplified from ACTB plasmid (kindly provided by Dr. E.G Stanley, Monash Immunology and Stem Cell Laboratories, Monash University, Australia) with primers: forward 5′ CACCCTTTCTAGAACTAGACT 3′ and reverse 5′ GTTAACCTCGACGTGAGCTGC 3′ and the resulting PCR product was cloned into pENTR/D-Topo vector. Human ubiquitinC (UbC) promoter was amplified from human genomic DNA using the following primers: forward 5′ GCCTCCGCGCCGGGTTTTGGC 3′ and reverse 5′ TCCACAACAAGAACCGCGAC 3′ and cloned into the pENTR/D Topo vector (Invitrogen).

Lentiviral vector stocks were produced by transient transfection of 293T cells with the p8.91 encapsidation plasmid [36], the VSV glycoprotein-G-encoding pHCMV-G plasmid [37], and the lentiviral recombinant vector as previously described [38]. Supernatants were treated with DNAseI (Roche Diagnostic) prior to ultracentrifugation and the resulting pellet was resuspended in Phosphate Buffered Saline, separated into aliquots and frozen at −80°C until use. The transduction efficiency of each vector stock was determined by FACS analysis as previously described [35].

### Lentiviral transduction

hESCs were transduced with vector particles harboring ACTB-, CMV-, EF1α-, PGK- and UbC-EGFP, respectively, at MOI 1 previously determined to generate low number of integrated viral vector copies. Briefly, 500000 hESCs were dissociated to single cells dissolved in 200 µl cell culture medium, incubated with virus at MOI 1 under gentle shaking for 1 h at 37°C, thereafter seeded on MEF cells in 2 ml cell culture medium as described above. Medium was changed the next day and cells were cultured to confluence. eGFP expressing cells were isolated using FACS sorting and cultured on Matrigel matrix (BD biosciences) in mTESR.1 medium (Stemcell Technologies). hESC line SA121 was transduced in three separate experiments and at different passages for eGFP expression in undifferentiated cells and EB differentiated cells.

### Flow cytometry

To isolate eGFPexpressing cells, trypsin dissociated cells were filtered through Filcon filter 50M (BD bioscieneces) to remove aggregated cell clumps and were sorted on FACSVantageSE DiVAOption (BD Biosciences) equipped with DiVa 5.0.3 software. Analysis was performed in FlowJo (Tree Star). Cells were collected in cell culture medium. Reanalysis of sorted cells reproducibly showed a high purity (>98%).

To analyze eGFP expression, transduced cells were dissociated to single cells and measured on a FACSCalibur equipped with CellQUEST software (BD biosciences). A population of 5000 cells was analyzed and 7-aminoactinomycin-D (7AAD) (Sigma) were used to exclude dead cells.

### Real time quantitative PCR

For quantification of copy number of integrated viral vectors, genomic DNA (gDNA) was extracted (Sigma GenElute Genomic Mammalian DNA Mini prep kit) from eGFP+ FACS sorted cells cultured without MEF cells. Quantification of *eGFP* was compared to a single copy reference gene *CDX2* using PCR cycling conditions 50°C for 2 min, 95°C for 2 min followed by 40 cycles, denaturation at 95°C for 15 sec, annealing at 60°C for 25 sec, and extension at 73°C for 30 sec. The correct PCR-products were confirmed by agarose gel electrophoresis (2% w/v) and melting curve analysis. *CDX2* was amplified with forward (fwd) 5′AGAGGGACTCAAGGGAAAGG′3 and reverse (rev) 5′ GGTCTGGGAAGGGAAGAGAA′3 primers and *eGFP* with fwd primer 5′ CTTGTACAGCTCGTCCATGCCG′3 and rev primer 5′AACATCGAGGACGGCAGCGT′3.

Briefly, *eGFP* and *CDX2* were amplified from plasmid and genomic DNA, respectively, followed by purification of PCR products. Serial dilution of *eGFP* and *CDX2* PCR products, mixed in 1∶1 molar ratio, were used for generation of standard curve by qPCR. Linear amplification of *eGFP* and *CDX2* verified that the 2^−ΔΔC^T method could be used to compare quantified PCR product of *eGFP* cDNA to the reference gene *CDX2* [39].

mRNA gene expression analysis including mRNA extraction, cDNA synthesis and qPCR amplification were performed on eGFP+ EB isolated by FACS sorting, as described in [40]. The following primers were used for amplification; *SOX17* fwd 5′AAGGGCGAGTCCCGTATC′3 and rev 5′TTGTAGTTGGGGTGGTCCTG′3, *ALBUMIN* fwd 5′GCAAGGCTGACGATAAGGAG′3 and rev 5′ TGGCTTTACACCAACGAAAA′3, *PPARγ* fwd 5′GCTGGCCTCCTTGATGAATA′3 rev 5′TTGGGCTCCATAAAGTCACC′3, *CD31* fwd 5′ CCTGTCTTTCAGCCTTCAGC′3 and rev 5′CGCCTGTGAAATACCAACCT ′3, *PAX6* fwd 5′GAACAGACACAGCCCTCACA′3 and rev 5′ATCATAACTCCGCCCATTCA′3 and *NESTIN* fwd 5′ AGCGTTGGAACAGAGGTTG′3 and rev 5′GCTGAGGGAAGTCTTGGAG′3.Ct values were normalized to GAPDH amplified with fwd 5′ GTTCGACAGTCAGCCGCATC′3 and rev 5′GGAATTTGCCATGGGTGGA′3 and plotted as relative mRNA expression. qPCR measurements were performed on three biological replicates, PCR-amplified as three technical replicates and plotted as standard deviation of the mean (± s.d.).

### Immunocytochemistry

Cells were washed once in PBS and fixed in 4% PFA for 15 min, washed three times in PBS, permeabilized in 0,25 TritonX-100 for 15 min and blocked in 5% skim milk (Sigma) in 0,1% Triton X-100 (block buffer) (BDH). Primary antibodies mouse-α-Oct 3/4 (1∶500) (SantaCruz), mouse-α-Nanog (1∶500) (Sigma) and mouse-α-hES-Cellect (1∶500) (Cellartis AB) were incubated in block buffer 4°C over night. As secondary antibody, Cy3 donkey-α-mouse (Jackson ImmunoResearch) was added in a 1∶1000 dilution in PBS for 2 h at room temperature. Cell nuclei were stained with DAPI (Sigma). Immunostained eGFP expressing cells were visualized with Nikon Eclipse TE 2000-U Axioplan 2 fluorescence microscope and AxioVision LE software (Zeiss).

## Supporting Information

Figure S1 Transduction efficiency in hESCs and determination of number of integrated eGFP copies. A. Initial titration of the viral vector particles (MOI) needed to transduce hESC line SA121 at low transduction efficiency, measured as eGFP+ cells by FACS analysis. B–E. Transduction of hESC line Hues-4 with pTRIP-ACTB-, CMV-, EF1α-, PGK-or UbC-eGFP lentiviral vectors. B. Transduction efficiency measured by FACS analysis. C. eGFP copy numbers were measured by qPCR of eGFP+ cell populations. (D) 10 days after transduction, eGFP+ and eGFP– cells were isolated by FACS sorting, referred to as day 0. Sorted eGFP+ cells were maintained as undifferentiated cells for 50 days and promoter activities were monitored by FACS analysis at day 0, 15, 30, and 50. E. Intensity of eGFP fluorescent signal detected by FACS analysis (*p≤0.04 students t'test). B–E. Data are shown as mean of three independent experiments. Error bars represent standard deviation of the mean (± s.d.). F. Standard curve for amplification by qPCR of *eGFP* and the reference gene *CDX2* used to determine the number of integrated eGFP copies in transduced hESCs. Results are shown as five technical replicates of each dilution of DNA.
(0.58 MB TIF)

Table S1 % eGFP+ cells of hESC line Hues-4 transduced with pTRIP-ACTB-, CMV-, EF1α-, PGK or UbC-eGFP lentiviral vectors. Data are shown as mean of three independent experiments ± s.d.
(0.03 MB DOC)
