# Supplementary figures and images for: Quantitative Comparison of Constitutive Promoters in Human ES cells
Source: PLoS One. 2010 Aug 26;5(8):e12413. doi: 10.1371/journal.pone.0012413 (PMC2928720; doi:10.1371/journal.pone.0012413)

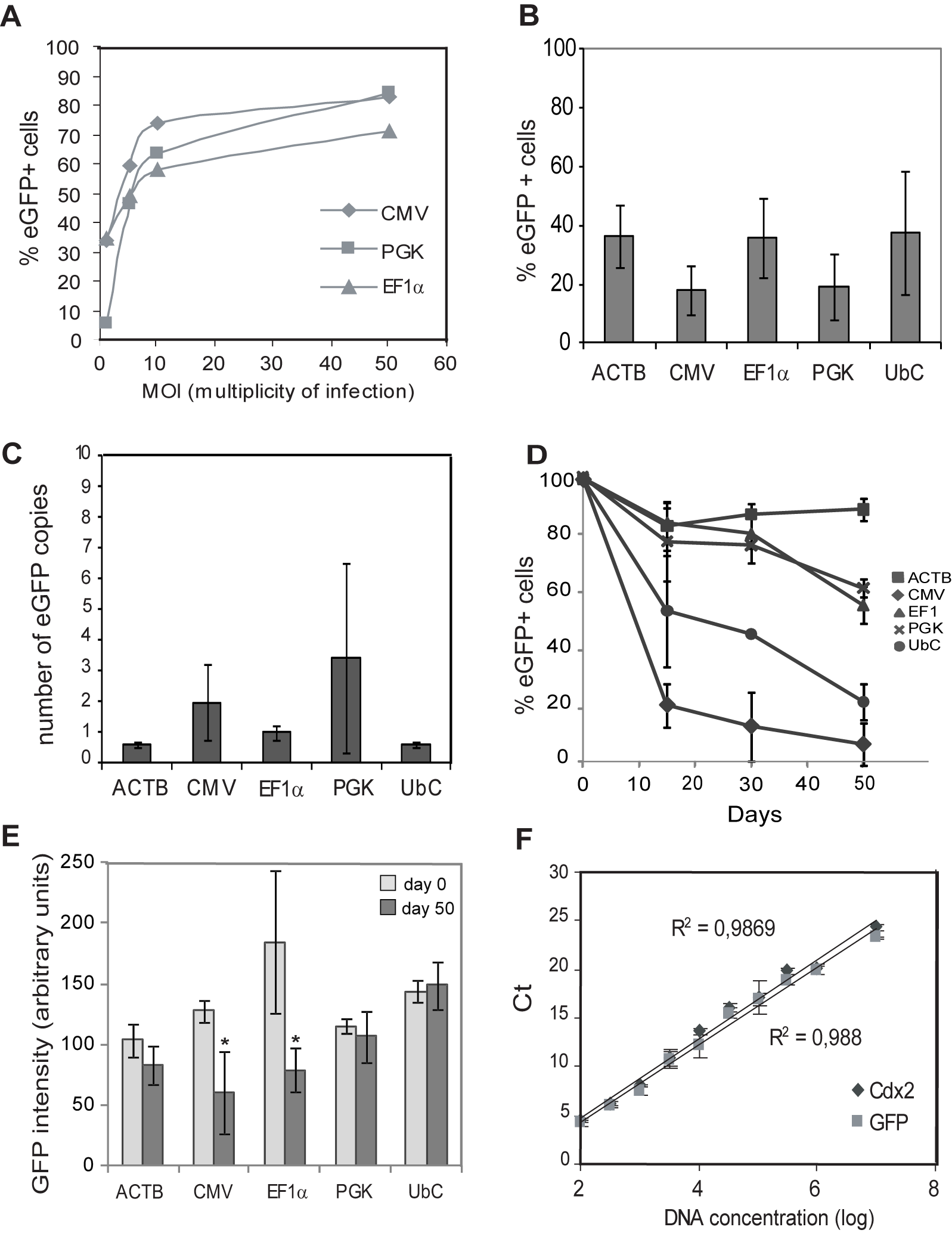

Supplement: Figure S1 — Transduction efficiency in hESCs and determination of number of integrated eGFP copies. A. Initial titration of the viral vector particles (MOI) needed to transduce hESC line SA121 at low transduction efficiency, measured as eGFP+ cells by FACS analysis. B–E. Transduction of hESC line Hues-4 with pTRIP-ACTB-, CMV-, EF1α-, PGK-or UbC-eGFP lentiviral vectors. B. Transduction efficiency measured by FACS analysis. C. eGFP copy numbers were measured by qPCR of eGFP+ cell populations. (D) 10 days after transduction, eGFP+ and eGFP– cells were isolated by FACS sorting, referred to as day 0. Sorted eGFP+ cells were maintained as undifferentiated cells for 50 days and promoter activities were monitored by FACS analysis at day 0, 15, 30, and 50. E. Intensity of eGFP fluorescent signal detected by FACS analysis (*p≤0.04 students t'test). B–E. Data are shown as mean of three independent experiments. Error bars represent standard deviation of the mean (± s.d.). F. Standard curve for amplification by qPCR of eGFP and the reference gene CDX2 used to determine the number of integrated eGFP copies in transduced hESCs. Results are shown as five technical replicates of each dilution of DNA. (0.58 MB TIF) [file pone.0012413.s001.tif]
