# Supplementary material for: Quantitative Comparison of Constitutive Promoters in Human ES cells
Source: PLoS One. 2010 Aug 26;5(8):e12413. doi: 10.1371/journal.pone.0012413 (PMC2928720; doi:10.1371/journal.pone.0012413)
Supplement: Table S1 — % eGFP+ cells of hESC line Hues-4 transduced with pTRIP-ACTB-, CMV-, EF1α-, PGK or UbC-eGFP lentiviral vectors. Data are shown as mean of three independent experiments ± s.d. (0.03 MB DOC) [file pone.0012413.s002.doc]

**Table S1.**

% eGFP+ cells of hESC line Hues-4 transduced with pTRIP-ACTB-, CMV-, EF1α-, PGK or UbC-eGFP lentiviral vectors. Data are shown as mean of three independent experiments ± s.d.

| **Days** | **ACTB** | **CMV** | **EF1**α | **PGK** | **UbC** |
| --- | --- | --- | --- | --- | --- |
| **0** | 98,7±0,6 | 98,6±1,5 | 99,0±1,0 | 99,3±1,1, | 98,7±1,1 |
| **15** | 82,6±8,4 | 21,3±7,4 | 83,8±5,0 | 77,2±13,5 | 53,5±18,9 |
| **30** | 86,5±0,1 | 14,0±11,8 | 80,0±10,1 | 76,0±1,0 | 45,6±0,42 |
| **50** | 88,2±3,8 | 7,8±7,3 | 55,5±6,3 | 61,3±3,0 | 22,4±6,3 |
